# Social Competence in Children with Borderline Intellectual Functioning: Delayed Development of Theory of Mind Across All Complexity Levels

**DOI:** 10.3389/fpsyg.2016.01604

**Published:** 2016-10-21

**Authors:** Gisella Baglio, Valeria Blasi, Francesca Sangiuliano Intra, Ilaria Castelli, Davide Massaro, Francesca Baglio, Annalisa Valle, Michela Zanette, Antonella Marchetti

**Affiliations:** ^1^IRCCS, Don Carlo Gnocchi Foundation OnlusMilan, Italy; ^2^Research Unit on Theory of Mind, Department of Psychology, Università Cattolica del Sacro CuoreMilan, Italy; ^3^Department of Human and Social Sciences, Università degli Studi di BergamoBergamo, Italy

**Keywords:** borderline intellectual functioning, social competence, Theory of Mind, executive functions, false belief

## Abstract

Borderline intellectual functioning (BIF) is characterized by heterogeneous cognitive difficulties, with an intelligence quotient (IQ) between 70 and 85 points, and a failure to meet the developmental and sociocultural standards for personal independence and social responsibility required in daily life. The fact that this population still remain a marginal clinical category, with no *ad hoc* diagnostic and therapeutic approaches, has stimulated the present research. Our goal was to study children with BIF investigating the development of Theory of Mind (ToM) as a pillar of social competence. Children with BIF (*N* = 28, 16 male/12 female, and mean age 9.46 ± 1.26 years) and children with typical development (TD; *N* = 31, 17 male/14 female; mean age 8.94 years ± 0.99) underwent a neurocognitive assessment and a ToM assessment. Children with BIF showed a significant lower performance across all the levels of ToM development investigated compared to the control group, and a correlation between executive functions and the advanced levels of ToM reasoning. These results constitute a first step in the direction of defining the clinical profile of children with BIF concerning ToM development, opening the way to future interventions in order to support the developmental evolution of this population in an adaptive direction.

## Introduction

Borderline intellectual functioning (BIF) is a controversial nosographic entity and a marginal clinical category that remains to be clearly defined (Cornoldi et al., 2014) and for which targeted diagnostics and therapeutic approaches do not exist.

Borderline intellectual functioning has been recently defined as a boundary status between disease and typical development, not stemming from a single neurodevelopmental syndrome and with heterogeneous functioning profiles (Salvador-Carulla et al., 2013). It is characterized by a wide range of cognitive difficulties, with an intelligence quotient (IQ) between 70 and 85 points, and a failure to meet the developmental and sociocultural standards for personal independence and social responsibility that affects daily activity (American Psychiatric Association, 2000, 2013; Tangney et al., 2004; Salvador-Carulla et al., 2013; Kok et al., 2016).

The lack of international consensus on diagnostic criteria makes it difficult to determine the current BIF prevalence. Some studies have reported a frequency ranging from 2% (Voigt et al., 2006) to 7–18% in the overall population (Ninivaggi, 2001; Salvador-Carulla et al., 2013), and around 7% among the school age population (Karande et al., 2008).

Typically, children with BIF show a heterogeneous neuropsychological profile (Salvador-Carulla et al., 2013; Peltopuro et al., 2014) including difficulties in executive functions, i.e., working memory, problem solving, and attention (Alloway, 2010; Schuchardt et al., 2010). The concern with this condition lies in its impact on the quality of everyday life of the affected subjects (Ninivaggi, 2001; Karande et al., 2008; Fernell and Ek, 2010; Salvador-Carulla et al., 2013). Indeed, this condition is closely associated with failure of social expectations, such as school dropouts, social isolation, inadequate affective relationships, and with the risk of developing mental illnesses in adolescence or adulthood, e.g., depression, suicidal ideation, self-harm, emotional distress, anxiety and antisocial disorders and, finally, future socio-economic disadvantage (Hassiotis et al., 1999, 2008; Douma et al., 2007; Emerson and Hatton, 2007; Ferrari, 2007; Karande et al., 2008; Emerson et al., 2010, 2011; Fernell and Ek, 2010; Peltopuro et al., 2014). A large part of these impairments, namely behavioral and affective disorders (Cook and Oliver, 2011; van Nieuwenhuijzen and Vriens, 2012), are an expression of a low social adaptive functioning and can be traced back to a lack of adequate complex socio-cognitive skills (Schalock et al., 2011).

A pivotal role in the social competences growth is played by Theory of Mind (ToM) or mentalization, i.e., the ability to attribute mental states (intentions, desires, emotions, and beliefs) to ourselves and others and to predict, on the basis of such inferences, our own and others’ behavior (Wimmer and Perner, 1983). An adequate level of ToM development and functioning has a cascade effect on other acquisitions, because ToM is involved in the increase of self-awareness, in the encoding of others’ behavior, in the self-regulation of emotions, in mastering novel problems and situation, in efficacious communication processes and, finally, in building satisfying relationships (Hughes and Leekam, 2004; Caputi et al., 2012; Ghrear et al., 2016). ToM evolves early in human development and seems to be strictly embedded with many cognitive and affective processes, such as frontal functions (Amodio and Frith, 2006; Grossmann, 2013; Devine and Hughes, 2014) emphatic concern (Ibanez et al., 2013) and affective syntonization (Xavier et al., 2013; Baimel et al., 2015), and attachment (Fonagy and Target, 1997). During development, ToM acquisition proceeds toward two levels of representation: abilities to construct ‘simple’ representations of another person’s mental states (as beliefs or thoughts, i.e., ‘What does John think?’) and more complex attributions (‘What does Paul think that Mary thinks?’). Learned in late childhood, the latter are considered to require more cognitive resources (for a review, see Miller, 2009), in particular competences executive functions (Leslie et al., 2005).

Given the relevance of ToM in social adaptive adjustment, several studies have investigated this competence in intellectual disability (ID) demonstrating a strong correlation between concrete reasoning and ToM impairments (Adrien et al., 1995; Baron-Cohen et al., 1999; Corcoran, 2001; Capozzi et al., 2009; Brojčin et al., 2014). Specifically, Schalock et al. (2011) highlighted a delay in the acquisition of the first and second order false belief reasoning—i.e., the ability to engage in complex levels of recursive thinking—in a sample of children with moderate ID. Moreover, Jervis and Baker (2004) discovered that children with non specific ID had better performance on ToM tasks compared to adults with equal intellectual capacity. This would be due to the increment in social isolation that occurs over the years and that is frequently associated with ID.

However, cognitive competences are necessary but not sufficient for ToM development. Despite the importance of these abilities in ToM development, a normal IQ can be associated with very poor performance in social skills and ToM tasks. For example, some subjects with high functioning autism (Baron-Cohen et al., 2001) remain strongly impaired in everyday social interactions despite high IQ profiles. This finding suggests that the link between IQ and ToM development deserves further consideration, especially in the population of children with BIF, in which the latter is associated with impairment in social skills.

On the basis of this evidence, we suppose that an inadequate ToM functioning is likely to be connected with a social impairment in the case of BIF. Given the lack of data about ToM in children with BIF and their risk to develop social disadvantages with age, we have explored this construct in this population in comparison with children with typical development (TD). Moreover, we explored the correlation between performance in an advanced ToM task and IQ. To this purpose, we used the Strange Stories task that strongly involves both cognitive functioning and social competences. Finally, given the importance of executive functions, in particular working memory, in ToM (Devine and Hughes, 2014) and in BIF, we investigated, in children with BIF, their connection with the performance in the Strange Stories task. Twenty-eight children with BIF and a group of 31 age-matched children with TD were studied through multiple ToM tasks and IQ measures.

## Materials and Methods

### Participants

Two groups of children, one with BIF and one with TD comparable for chronological-age and gender were included. All participants attended mainstream primary schools, near Don Gnocchi Foundation, and showed middle socio-economic status.

Twenty-eight children with BIF (16 male/12 female, mean age of 9.46 ± 1.26 years), with IQ ranging from 70 to 85, were enrolled in the study. A clinical interview revealed learning and social difficulties in all children with BIF. They were recruited from the Adolescence and Pediatric Neuropsychiatry Unit of our institution.

As control group 31 healthy students with TD (17 male/14 female; mean age of 8.94 years ± 0.99) and no history of neurological, psychiatric or systemic disease nor learning disability were enrolled through selection from the mainstream schools.

All children included in the study had never taken medications and underwent a clinical evaluation in order to exclude genetic syndromes, i.e., Down syndrome or X Fragile syndrome, and/or major neuropsychiatric problems, such as autism spectrum disorder or attention deficit hyperactivity disorder, other neurological conditions (epilepsy and traumatic brain injury), malformations, or systemic diseases, such as diabetes or immune disorders, and infectious disease involving the central nervous system.

The present study was approved by the scientific and ethics committees of (Don Gnocchi Foundation and Catholic University of the Sacred Heart). Parents gave written informed consent for participation in the study.

### Neuropsychological Assessment

All children were screened for general intelligence with the Wechsler Intelligence Scale for Children-III (Wechsler, 1991; Orsini and Picone, 2006).

Within their routine clinical evaluation, children with BIF underwent a neuropsychological assessment of executive functions which included: Semantic and Phonological Fluency (from the Neuropsychological Assessment Battery for children: BVN 5-11; Bisiacchi, 2005); the Working Memory Index (WMI from WISC-IV; Wechsler, 2003; Orsini et al., 2012) that included the subsequent subtest digit span and letter-number sequencing; modified Bells test accuracy and rapidity (Biancardi and Stoppa, 1997) a barrage task that evaluates immediate and sustained selective attention; Tower of London (TOL; Sannio Fancello et al., 2006) a task to measure planning ability and inhibitory control.

All these tests are standardized and their psychometrics properties are illustrated in their respective manuals and references.

### ToM Assessment

All participants were tested with a paper-pencil ToM battery in order to assess their level of mentalizing development. For a more sensitive measurement of ToM ability, the battery consisted of a first order false belief task (Deceptive Box Task; Perner et al., 1987); two second order false belief tasks, Look-prediction task (Astington et al., 2002; Antonietti and Giorgetti, 2006; Castelli et al., 2014) and Ice-cream story (Perner and Wimmer, 1985; Marchetti et al., 2014) and advanced ToM tasks, i.e., a selection of four Strange Stories and their control task, the Physical Stories (Happé, 1994; Happé et al., 1998; Mazzola, 2002). Each task provides for one or more control questions to test the understanding of the logical order and the explicit elements in the story without inferring mental states. These tools are designed as research assessments and are widely used to investigate this construct in a life-span perspective (Fonagy and Allison, 2012). Meta-analyses confirm the validity of the ToM battery used in this study both in typical (Wellman et al., 2001; Henry et al., 2013) and atypical population (Stewart et al., 2016).

The first order false belief task consists of a real experience of a false belief. A closed candies box, whose content has been secretly substituted with an unexpected object, is shown to the participants who are asked to say what it contains. After the discovery of their own false belief, the box is closed again and the investigator asks them to predict what another person would say when shown the closed box. The participants are asked three control questions and two test questions regarding the false belief, the first concerning that of another person, and the second for one’s own false belief. 1 point is assigned in the case of a correct answer, whereas 0 in the case it was incorrect.

The second order false belief tasks are more complex tasks, suitable to test a higher level of mentalistic reasoning. Through the Look-prediction task it is possible to evaluate the acquisition of the second order false belief reasoning because the situation that is presented to the participants requires high order, recursive mentalistic reasoning. The story is told and the participants have to predict where the co-protagonist, on the basis of his/her false belief, would think that the other person will look for the object. The main elements of the story are illustrated with pictures that remain available during the whole interview in order to reduce as much as possible the memory load. In the same way, the Ice cream story, the classical second order false belief task, explores the abilities to manage the mental contents employed in the story. For the Look-prediction five questions are asked, two of which are used as controls, one as an explanation, and the remaining two are the actual test questions. The Ice-cream story, included three control questions, one explanation question and one test question. In both tasks 1 point is assigned in the case of correct answer, whereas 0 in the case it was incorrect.

The Strange Stories task measures a higher level of ToM because of their complexity and structure. Each story describes a daily situation and refers to a specific mental content i.e. double bluff, white lie, misunderstanding and persuasion. Participants have to decode not only the mental content but also the dynamic of the story in a single and coherent view (see Castelli et al., 2011 for a complete description of this ToM battery). For each story there is one control question (except for the double bluff story, which has two) and one test question. All the tasks described were chosen as they have been shown to be sufficiently reliable and valid measures of ToM competences for children in the age range included in this study (Happé, 1994; Wellman et al., 2001). 0 point is assigned in the case of incorrect answer, 1 in the case the answer was incomplete or partially correct, finally 2 for a full and complete answer. In addition, it is possible to code if subjects use mental state term (MS, e.g., thinks, knows, wants, etc.) or if an inappropriate mental state is attributed (MSx). A recent study (Devine and Hughes, 2016) showed the reliability and the validity in childhood of Strange Stories.

The whole battery was administered individually; answers were audio-recorded and then coded once the session was closed.

### Procedure

Children with BIF were tested at Don Gnocchi foundation whereas children with TD at school. All participants were individually tested in a quiet room in two sessions: the first for the WISC-III and the second for the ToM battery. This was administered with increasing complexity sequence.

### Data Analysis

Statistical analyses were conducted with MedCalc software (v14.8.1). Demographic and intellectual functioning differences between groups were tested using the *t*-test or χ^2^ test as appropriated.

Theory of Mind data were screened based on a conservative inclusion criteria: for each participant, only the stories in which the control questions were answered correctly were included in the analyses. The scoring of each story included only the test and the explanation questions. Control questions were only used as inclusion criteria and thus were excluded from the total score.

The two groups were compared by ANCOVA analyses with age as covariate of no interest, using a General linear model (GLM) to explore differences in the following variables: performance at first order false belief task (Deceptive box, score 0 to 2); at second order false belief task (Ice cream story and Look-prediction, score ranging from 0 to 6); Strange Stories (score ranging from 0 to 8) and Physical Stories (score ranging from 0 to 8). Furthermore, to test relationship between performance in advanced ToM task (Strange Stories) and IQ Pearson’s correlation analyses were performed: Strange Stories and WISC-III score; Strange Stories and executive functions tests (semantic and phonological fluency, WMI) within the two groups (TD and BIF). Moreover, to explore the link between advanced ToM task (Strange Stories) and executive functions we performed an additional correlation analysis.

## Results

### Group Differences in Age, Gender, and IQ

Children with BIF were not significantly different from children with TD on the matching variables of age and gender (**Table 1**). Performance on the WISC-III was significantly different between the two groups as expected as the inclusion criteria were also based on IQ (**Table 1**).

**Table 1:** Means and standard deviations (*SD*) of the sample’s demographics and performance at WISC III.

	BIF	TD	Group comparison (*p*-value)
*N*	28	31	
Mean age (*SD*)	9.46 (1.26)	8.94 (0.99)	n.s.^∗^
Gender (M:F)	16:12	17:14	n.s.^#^
FSIQ	78.61 (3.87)	105.36 (10.38)	*p* < 0.001^∗^
VIQ	80.28 (4.93)	101.48 (10.36)	*p* < 0.001^∗^
PIQ	81.93 (6.15)	107.93 (11.46)	*p* = 0.002^∗^


*Children with BIF, borderline intellectual functioning; children with TD, typical development; FSIQ, full scale intelligence quotient; VIQ, verbal IQ; PIQ, performance IQ. n.s., not statistically significant ^∗^*t*-test; ^#^χ ^2^ test.*

**FIGURE 1:**
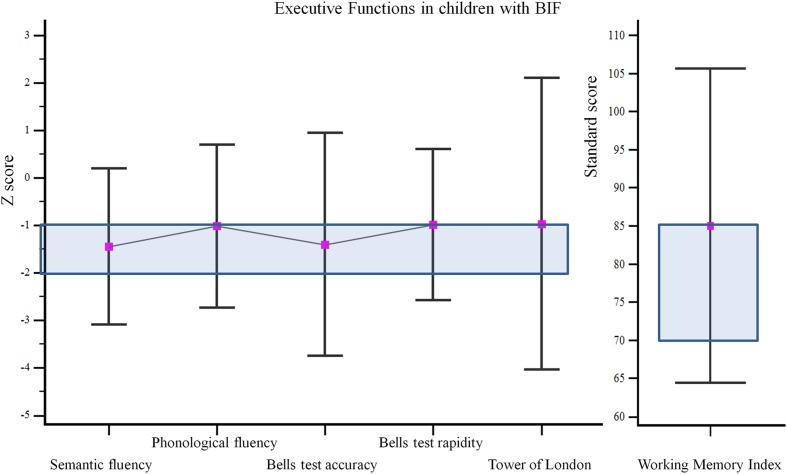
**Results of the neuropsychological assessment of executive functions of children with borderline intellectual functioning (BIF).** The colored box indicates performance in the borderline range, i.e., between -1 and -2 *Z*-scores.

Results of the neuropsychological assessment of executive functions of children with BIF are shown in **Figure 1**: performances were in the borderline range in all tests.

### Group Differences in ToM Performance

Regarding the performance at the ToM tasks, the two groups of children performed similarly with respect to the control questions. This means that the two groups were equal in the capacity to understand the concrete contents of the story and to remember the main information. Concerning the false belief tasks, the statistical analysis between children with TD and children with BIF highlighted a significant difference for the first order total score index of the deceptive box task (ANCOVA *p* < 0.05; **Table 2**) and for the second-order Look-prediction task, and no difference for the second-order Ice-cream task. For the Ice-cream task, fewer participants passed the filter of the control questions (only 21 children with TD and 16 with BIF), thus showing that this story was more difficult to understand in its contents, besides the false beliefs inferences, for both groups (**Table 2**).

**Table 2:** Comparison of the ToM abilities between the two groups of children.

ToM competence	*N* participants (TD/BIF)	ToM task	BIF (mean ±*SD*)	TD (mean ±*SD*)	*p*-Value (Cohen’s *d*)
I order FB	58 (31/27)	Deceptive Box_Total score	1.89 ± 0.32	2.00 ± 0.00	0.037 (0.55)
II order FB	56 (30/26)	Look-prediction total score	1.15 ± 0.97	2.13 ± 1.07	0.001 (1.024)
	37 (21/16)	Ice-cream total score	0.56 ± 0.89	1.09 ± 1.34	0.106 (0.542)
Advanced	56 (31/25)	Strange Stories total score	3.71 ± 2.07	5.09 ± 2.15	0.008 (0.757)
Control	59 (31/28)	Physical Stories total score	3.61 ± 1.55	4.22 ± 2.14	0.132 (0.371)


*TD, typically developing children; children with BIF, borderline intellectual functioning; FB, false belief; () denotes number of participants included in the comparison according to their performance at the control questions.*

Regarding the advanced ToM tasks, we found significantly lower performance of children with BIF compared to children with TD in the Strange Stories (*p* < 0.05), with no differences in the performance on the Physical Stories tasks (**Table 2**).

### Correlation between Intellectual Functioning and ToM Competences

To investigate the role of intellectual functioning in ToM abilities, we performed a correlation analysis between Strange Stories and WISC III scores in children with TD.

**FIGURE 2:**
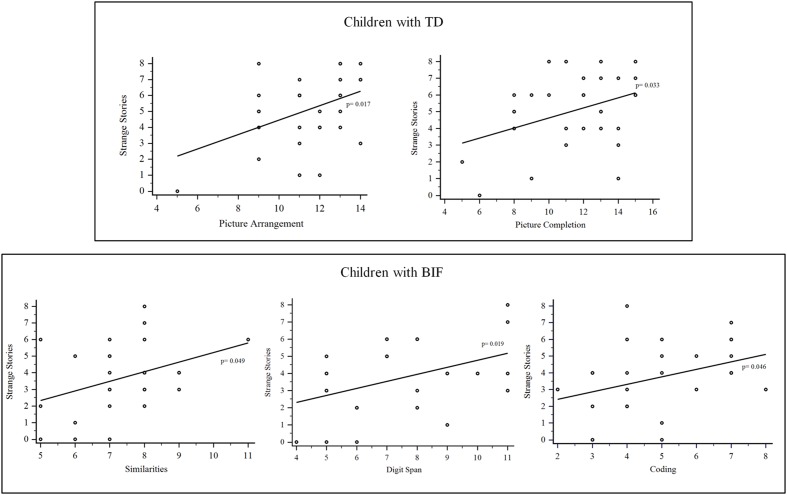
**Results of the correlation between Strange Stories and WISC-III subtests in children with typical development (TD) and BIF**.

Results showed significant correlations only between the Strange Stories task and two WISC-III subtests: Picture completion (*p* < 0.05) and Picture Arrangement scores (*p* < 0.05) (**Figure 2**; **Table 3**).

**Table 3:** Pearson’s correlations between performance at the Strange Stories task and Wisc-III scores.

		Strange Stories	
		
		TD	BIF
FSIQ	Correlation coefficient	0.20	0.35
	*p-*value	0.28	0.07
VIQ	Correlation coefficient	0.20	0.15
	*p-*value	0.28	0.46
PIQ	Correlation coefficient	0.13	0.28
	*p*-value	0.50	0.15


*Children with TD, typical development; children with BIF, borderline intellectual functioning; FSIQ, full scale intelligence quotient; VIQ, verbal IQ; PIQ, performance IQ.*

To determine the impact of borderline intellectual functioning in ToM abilities we performed the same correlations as above in children with BIF. A significant correlation was found only between the Strange Stories task score and Similarities (abstract reasoning; *p* < 0.05), Coding (*p* < 0.05), and Digit Span scores (*p* < 0.05; **Figure 2**). **Table 3** summarizes the correlation analysis between the Strange Stories task and IQ scores.

### Correlation between Executive Functions and ToM Performace in Children with BIF

To test the relation between ToM abilities and executive functions we performed a correlation between Strange Stories and the fluency tests and Working Memory Index. Results showed a significant correlation between Strange Stories performance and semantic fluency and Working Memory Index, and a tendency toward the significance with phonological fluency (**Table 4**).

**Table 4:** Correlation between the Strange Stories task and executive functions in children with BIF.

		Strange Stories
Semantic Fluency	Corr coeff	0.44
	*p-*value	0.02
Phonemic Fluency	Corr coeff	0.37
	*p-*value	0.06
Working Memory Index (WISC IV)	Corr coeff	0.47
	*p-*value	0.05


*BIF, Borderline intellectual functioning; Corr coeff, correlation coefficient.*

## Discussion

The principal aim of this study was to determine if children with BIF, characterized by a low IQ and difficulties to meet developmental and socio-cultural standards, show a pattern of ToM abilities which deviates from the one observed in typically developing children.

The first result that we found was the absence of significant differences between the two groups in the control questions of the ToM tasks. This demonstrates that all participants were able to overcome the task when mental contents were not included in the reasoning, presenting a comparable instruction comprehension and understanding of the cause–effect relationship of the facts narrated in the stories. Indeed, in these exercises, participants were required to understand the logical order of the events and to answer questions only on the basis of the elements that are explicit in the story, i.e., without inferring mental states.

However, significant differences between the two groups were found when children were required to consider the characters’ mental states. This was true across all ToM tasks, from the simplest one, the first order false belief, to the more advanced ones, the Strange Stories. In fact, children with BIF performed lower than children with TD at Deceptive Box, a task that can be considered as the baseline of ToM reasoning, as it evaluates first-order false belief, and it is usually overcome around 4 years of age (Wimmer and Perner, 1983). In this task, both short-term memory and the ability to decentralize one’s own point of view are stimulated; the comprehension of the task is made easier by the participants’ direct experience of the real situation. Nevertheless, our data show that children with BIF did not perform at the expected developmental standards. In addition, children with BIF performed lower than children with TD in the second order false belief tasks, which evaluate a more difficult level of recursive thinking. This aspect of ToM is usually successfully managed around 7–9 years of age (Perner and Wimmer, 1985). The story is more complex than the first order false belief one, with an increasing number of agents engaged in embedded mental states. Children with BIF were not able to distinguish what each character actually knew from their own omniscient knowledge. In all these stories, characters have only incomplete or partial information about the events, as happens in daily life. It is from this lack of information that the false belief reasoning takes place. The participants should be able to handle such absence by incorporating the other’s beliefs. In order to do that, they must identify and keep in mind all the relevant information to have various prospects at the same time and manage the situation. Finally, differences between the two groups were found also in the advanced ToM tasks, where multiple cognitive and affective abilities are strictly involved (Happé, 1994). Events are put into a social context, where the understanding of the protagonists’ behavior requires an affective syntonization, the assumption of the character’s intentions, the anticipation, and prediction of others possible behaviors. These results seem to indicate a delay in ToM abilities development. This hypothesis deserves future investigation with a control group matched for mental age.

Because ToM, in particular advanced ToM ability, is closely embedded with cognitive competences, we explored the impact of global intelligence on this complex process. We looked into the specific intellectual functioning of each group to determine how this interacted with advanced ToM tasks such as the Strange Stories. Results showed a positive correlation with the Picture Completion and the Picture Arrangement subtests in children with TD. These two tasks require rapid visual-perceptive analysis and processing. To be effectively solved, they necessitate the use of global cognitive strategies for a rapid and complete analysis of all the elements rather than an analytical approach that focuses on perceptual details (Vakil and Lifshitz-Zehavi, 2012). Indeed, the latter is more time demanding. Moreover, in our opinion, the Picture Arrangement subtest of the WISC-III requires a large degree of ToM to be accomplished: some stories require irony and sense of humor to understand intentions of the others or consequences of someone’s actions or chain of events. On the other hand, the Picture Completion requires the ability to anticipate the object’s mental image to identify its main elements and therefore the missing one, a skill highly involved in ToM tasks.

Differently, children with BIF showed a pattern of positive correlations with Similarities, Coding and Digit Span. These subtests assess short-term and working memory (Digit span and Coding), inhibitory control and novel material learning ability (Coding) and verbal fluid reasoning (Similarities), all abilities that engage functions connected with the frontal lobe (executive functions and FI). Moreover, we found that performance of children with BIF at executive function tests were in the borderline range and correlated (verbal fluency and working memory index) significantly with the Strange Stories task scores. Many studies demonstrated that children with BIF have difficulties in executive functions (Henry and MacLean, 2002; van der Meer and van der Meere, 2004; Van der Molen et al., 2007, 2009, 2014; Bonifacci and Snowling, 2008; Alloway, 2010; Hartman et al., 2010; Henry and Winfield, 2010). Our explanation is that our results may be due to the poor executive functions of children with BIF leading to an overload of information with the increasing of the complexity of the tasks. Poor executive functions could affect the capacity to keep online the main information of the story (for example the characters or their actions) while they select and handle those necessary to master the situation. The hypothesis of an overload of data could, also, underpin the preference of children with BIF for analytic analysis, as highlighted in another study (Vakil and Lifshitz-Zehavi, 2012).

The connection between ToM abilities and executive function that we found is also supported by several studies (see for a review, Devine and Hughes, 2014). An important aspect of executive functions relates to the ability to constantly update personal knowledge with new information, to learn by experience and master real life situation adaptively and to be socially competent. Recent studies have demonstrated that psychosocial adaptation is also connected to fluid intelligence (FI; Huepe et al., 2011), defined as the ability to reason and solve new problems independently from the acquired knowledge (Cattell, 1963; Cattell, 1967; Duncan, 2005; for a review see Nisbett et al., 2012). Executive functions and FI are strictly embedded; in fact FI is also important to modulate the ToM as demonstrated in experiments with the Eyes Test, which is an advanced ToM task (Ibanez and Manes, 2012; Ibanez et al., 2013; Baker et al., 2014). FI and executive functions are, indeed, engaged not only in cold information processing, such as logical and abstract reasoning, but also in hot processing, such as social cognition. This confirms the cross-influences existing between the cognitive and the social-affective processes (Liverta Sempio and Marchetti, 1995).

In our study, the positive correlation between the Strange Stories task and the Similarities that we found in children with BIF can be connected not only with FI but also with a deficit in meta-representational competences, as demonstrated in other studies involving children with mild to borderline intellectual disabilities (Karmiloff-Smith et al., 1995; Melogno and Becciu, 1999; Ivancich Biaggini, 2004; Capozzi et al., 2009). Indeed, ToM requires meta-representational abilities, such as lower-order representations (i.e., representations of reality) and higher-order representations (i.e., the ability to form thoughts about attributed thoughts).We suppose that when there is an overload of information to be processed, children with BIF face difficulty with both types of meta-representations that are necessary for both tasks: Strange Stories and Similarities.

Moreover, our results showed that children with BIF perform poorly on all false belief tasks, even the easiest ones. These children seem to be still dealing with the belief-desire psychology stage, which is an early (around 3–4 years of age) evolution step. At this level children understand that actions are driven by desires, thought, intention and true belief but they are not able to comprehend that behavior can also be justified by misinterpretation, i.e., false belief.

## Conclusion

The results of our study show that children with BIF have a deficit in ToM that is strictly connected with their executive functions and meta-representation competences. These results can be important to develop treatment strategies able to support these children in their social skills during childhood and to prevent the social disadvantages that they might face in adulthood.

Many evidences from the literature prove that ToM is not an all-or-nothing process, but rather it is a set of skills that people continue to develop during the whole life (Fonagy and Allison, 2012). Nevertheless, we are aware that “ToM is sometimes necessary, but never sufficient”, to quote Astington (Astington, 2003) and that future studies are needed to explore the impact of ToM in everyday social life of children with BIF.

The results suggest that an early intervention is essential to support the mentalization development (Fabio et al., 2009, 2013) as already demonstrated in other disabilities (Fonagy and Allison, 2012). In the specific case of children with BIF, the timing of the intervention is crucial. Many studies have shown that the “window of opportunity” is particularly open and fruitful during childhood (Alexander and Entwisle, 1988; Alexander et al., 1995), as brain plasticity (see a Dennis et al., 2013 for a review) and the opportunity to positively influence the developmental course are increased (Pianta and Walsh, 1996). Furthermore, recent studies have demonstrated a cerebral cortical developmental delay in children with BIF (Baglio et al., 2014). According to these evidences, it is essential to better understand the functioning difficulties of children with BIF. This will allow for the development of targeted strategies to minimize the gap with their peers as soon as possible and to redirect these children toward a typical developmental pattern.

## Author Contributions

All authors have reviewed critically the work, provided important intellectual content and approved the final form.

GB, VB, and FB designed the research, analyzed data, interpreted the results and drafted the manuscript; GB also performed neuropsychological and ToM evaluation; MZ recruited patients and analyzed the clinical data. FS, IC, DM, AV, and AM have dealt with supplying ToM tasks material, analyzing ToM data and drafted the manuscript.

## Conflict of Interest Statement

The authors declare that the research was conducted in the absence of any commercial or financial relationships that could be construed as a potential conflict of interest.
